# Prognostic impact of *ACTN4* gene copy number alteration in hormone receptor-positive, HER2-negative, node-negative invasive breast carcinoma

**DOI:** 10.1038/s41416-020-0821-y

**Published:** 2020-04-08

**Authors:** Teppei Sugano, Masayuki Yoshida, Mari Masuda, Makiko Ono, Kenji Tamura, Takayuki Kinoshita, Hitoshi Tsuda, Kazufumi Honda, Akihiko Gemma, Tesshi Yamada

**Affiliations:** 1 Division of Chemotherapy and Clinical Research, National Cancer Center Research Institute, Tokyo, 104-0045 Japan; 2 Department of Pulmonary Medicine and Oncology, Graduate School of Medicine, Nippon Medical School, Tokyo, 113-8602 Japan; 3 Department of Pathology and Clinical Laboratories, National Cancer Center Hospital, Tokyo, 104-0045 Japan; 4 Department of Medical Oncology, Cancer Institute Hospital, Japanese Foundation for Cancer Research, Tokyo, 135-8550 Japan; 5 Departments of Breast and Medical Oncology, National Cancer Center Hospital, Tokyo, 104-0045 Japan; 6 Department of Breast Surgery, National Cancer Center Hospital, Tokyo, 104-0045 Japan; 7 Department of Basic Pathology, National Defense Medical College, Saitama, 359-8513 Japan; 8 Department of Biomarkers for Early Detection of Cancer, National Cancer Center Research Institute, Tokyo, 104-0045 Japan

**Keywords:** Prognostic markers, Tumour biomarkers, Prognostic markers, Tumour biomarkers

## Abstract

**Background:**

Most patients with hormone receptor (HR)-positive, human epidermal growth factor receptor type 2 (HER2)-negative breast cancer can be cured by surgery and endocrine therapy, but a significant proportion suffer recurrences. Actinin-4 is associated with cancer invasion and metastasis, and its genetic alteration may be used for breast cancer prognostication.

**Methods:**

The copy number of the actinin-4 (*ACTN4*) gene was determined by fluorescence in situ hybridisation (FISH) in two independent cohorts totalling 597 patients (336 from Japan and 261 from the USA) with HR-positive, HER2-negative, node-negative breast cancer.

**Results:**

In the Japanese cohort, multivariate analysis revealed that a copy number increase (CNI) of *ACTN4* was an independent factor associated with high risks of recurrence (*P* = 0.01; hazard ratio (HR), 2.95) and breast cancer death (*P* = 0.014; HR, 4.27). The prognostic significance of *ACTN4* CNI was validated in the US cohort, where it was the sole prognostic factor significantly associated with high risks of recurrence (*P* = 0.04; HR, 2.73) and death (*P* = 0.016; HR, 4.01).

**Conclusions:**

Copy number analysis of a single gene, *ACTN4*, can identify early-stage luminal breast cancer patients with a distinct outcome. Such high-risk patients may benefit from adjuvant chemotherapy.

## Background

In most developed countries, the breast cancer mortality rate has declined over the last few decades^1^ owing to the development of new screening, diagnostic and therapeutic modalities. However, breast cancer is a heterogeneous disease with a wide spectrum of biological behaviour, and a significant proportion of patients still suffer recurrences and die of the disease. On the basis of intrinsic molecular characteristics, breast cancer is classified into at least four subtypes: the HER2-enriched, basal-like, luminal A, and luminal B (HER2-positive and -negative) subtypes.^2^ This classification is closely associated with various pathological features, response to therapeutics and prognosis.

The luminal A subtype typically expresses a high level of oestrogen receptor (ER)/progesterone receptor (PgR) and is histologically low-grade, characterised by low cell proliferation. Luminal B tumours are ER-positive, but may have variable degrees of ER/PgR expression; histologically, they are high-grade and have high cell proliferation.^3^ Approximately 20% of luminal B tumours are HER2-positive and treated with anti-HER2 therapeutics, but patients with luminal B/HER2-negative tumours need to be accurately diagnosed and receive adjuvant chemotherapy. Luminal tumours were originally defined by gene expression profiling,^4^ but the distinction between luminal A- and B-like tumours is most commonly made by Ki-67 immunostaining. However, problems such as inter-observer or inter-institutional variation and lack of an optimal cut-off value make Ki-67 immunostaining unreliable.^5,6^ Several commercially available multi-gene assays including the 21-gene recurrence score (Oncotype DX) and 70-gene signature (MammaPrint) assays are well standardised and more accurate for predicting the outcome of axillary node-negative luminal breast tumours.^7^ However, these multi-gene assays are expensive,^8^ and the development of a more cost-effective single gene assay would be desirable.

We originally identified actinin-4 as an actin-binding protein closely associated with cell motility and cancer invasion.^9^ Forced expression of actinin-4 increased cell motility and promoted metastasis, whereas knockdown of actinin-4 suppressed the invasiveness of cancer cells.^9–13^ Amplification of the *ACTN4* gene was detected in a small (8–16%) but substantial subset of stage I (node-negative) lung adenocarcinomas with a markedly unfavourable outcome.^14^ We and others have shown that amplification/overexpression of the *ACTN4* gene/actinin-4 protein is a significant prognostic factor associated with poor outcome in patients with colorectal,^15^ pancreatic,^11^ ovarian,^16,17^ salivary gland^18^ and high-grade neuroendocrine pulmonary tumours.^19^

Luminal B breast cancer has a unique profile of gene copy number alterations.^2^ Gene amplifications and other gross chromosomal aberrations are frequently detected in luminal B tumours. We have hypothesised that an increase in the copy number of the *ACTN4* gene might also identify luminal B-like/HER2-negative breast cancers with a high risk of recurrence. Here we report for the first time that *ACTN4* can be used as a highly specific single genetic biomarker to identify high-risk patients with HR-positive, HER2-negative, node-negative invasive breast cancer.

## Methods

### Patients and tissue microarrays

**Fig. 1:**
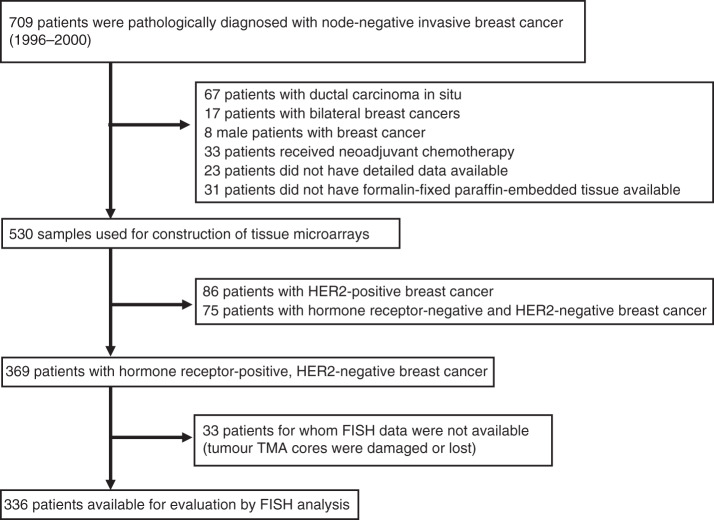
Selection of eligible individuals (Japanese cohort).

The use of human samples for this study was reviewed and approved by the Institutional Review Board of the National Cancer Center (Tokyo, Japan). We constructed tissue microarrays (TMAs) from formalin-fixed, paraffin-embedded tissue blocks that had been prepared for routine pathological diagnosis of 709 primary breast cancers surgically resected at the National Cancer Center Hospital (NCCH) (Tokyo, Japan) between 1996 and 2000 and pathologically diagnosed as node-negative, using a tissue microarrayer (Azumaya, Tokyo, Japan) as described previously.^6^ The status of ER, PpR, HER2 and Ki-67 was determined by immunohistochemistry and FISH, as described previously.^6^ Histological and nuclear grading was performed as described previously.^6^ Among the 709 cases, there were 369 HR-positive, HER2-negative invasive breast cancers (Fig. 1). Pathological staging was performed according to the Unio Internationalis Contra Cancrum (UICC) Tumor-Node-Metastasis (TNM) classification (7th edition, 2009).

We obtained a set of 10 TMAs containing 550 tissue cores of node-negative breast cancers (Breast Stage I Prognostic TMA) from the Cooperative Human Tissue Network (CHTN) through the Cancer Diagnosis Program (CDP) of the National Cancer Institute (NCI).^20^ The TMAs included 330 patients with HR-positive, HER2-negative, node-negative invasive breast cancer (Supplementary Fig. S1**)**.

### FISH

**Fig. 2:**
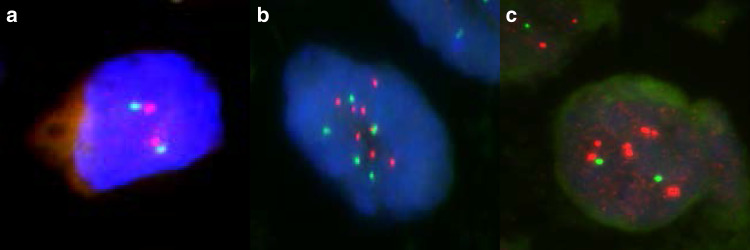
FISH analysis of the *ACTN4* gene. Representative cases of NCN (**a**), gene amplification (**b**) and high polysomy (**c**). Nuclei were stained with DAPI.

Bacterial artificial chromosome (BAC) clones containing the *ACTN4* gene and centromere of chromosome 19 were isolated as described previously,^14^ and Good Manufacturing Practice (GMP)-grade FISH probes were prepared by Abnova (Taipei, Taiwan). TMAs were hybridised with the FISH probes at 37 °C for 48 h. The nuclei were counterstained with 4,6-diamidino-2-phenylindole (DAPI). The numbers of fluorescence signals of *ACTN4* (red) and centromere (green) in the nuclei of 20 interphase tumour cells were counted by an investigator blinded to the clinical data. The FISH patterns with gene amplification (≥2.0-fold increase relative to the centromere) and high polysomy (≥5 mean copies per cell) were defined as having an *ACTN4* copy number increase (CNI), and all others were defined as having a normal copy number (NCN) according to the standard Colorado criteria^21^ (Fig. 2).

### Statistical analyses

The statistical significance of correlations was examined using Fisher’s exact test. Breast cancer-specific survival (BCSS) was measured as the interval from surgery to the date of breast cancer death or last follow-up. Disease-free survival (DFS) was defined as the length of time from surgery to the first detection of new lesions. BCSS and DFS were estimated by the Kaplan–Meier method and compared by log-rank test. Univariate and multivariate analyses were carried out using the Cox regression model. All statistical analyses were performed with EZR (Saitama Medical Center, Jichi Medical University, Saitama, Japan), a graphical user interface for R (The R Foundation for Statistical Computing, Vienna, Austria).^22^ Differences were considered to be significant at *P* < 0.05.

## Results

### Patient characteristics

As the TMAs had been used in our previous study,^6^ several tissue cores had worn out or peeled off from the slides. Among the 369 HR-positive, HER2-negative, node-negative invasive breast cancers in the Japanese cohort, 336 samples were evaluable by FISH. A total of 36 recurrences and 13 deaths from breast cancer occurred during the median follow-up period of 118.1 (range, 0.6–160.3) months. The age of the patients ranged from 27 to 85 years (median 57 years). Other clinicopathological characteristics of the patients are shown in Table 1.

**Table 1:** Correlation of ACTN4 gene status with clinicopathological characteristics (Japanese cohort).

Characteristics	Total	*ACTN4* FISH		*P* value*
		NCN	CNI	
Total	336	313	23	
Age				
≤50	130	122	8	0.826
>50	206	191	15	
Menopausasl status				
Premenopause	135	127	8	0.664
Postmenopause	201	186	15	
Area of invasion tumour				
≤2.0	249	232	17	1
>2.0	87	81	6	
Histology				
Invasive ductal carcinoma	277	256	21	0.393
Others	59	57	2	
Histological grade				
1 or 2	255	241	14	0.079
3	79	70	9	
Unknown	2	2	0	
Nuclear grade				
1 or 2	236	224	12	0.06
3	100	89	11	
Lymphovascular invasion				
Negative	201	189	12	0.51
Positive	135	124	11	
Ki-67				
<14%	191	180	11	0.39
≥14%	145	133	12	
Adjuvant chemotherapy				
Yes	136	125	11	0.512
No	200	188	12	
Hormone therapy				
Yes	166	154	12	0.072
No	169	159	10	
Unknown	1	0	1	

*FISH* fluorescence in situ hybridisation, *NCN* normal copy number, *CNI* copy number increase. *Fisher’s exact test.

Among the 336 cases, high polysomy was detected in 6 patients (1.7%) and gene amplification was evident in 17 patients (5.1%). A total of 23 patients (6.8%) were defined as having *ACTN4* CNI, and the remaining 313 patients (93.2%) were defined as having NCN. *ACTN4* gene status was not significantly associated with age, menopausal status, area of invasive tumour, histology, histological grade, lymphovascular invasion or Ki-67 status (Table 1).

### Prognostic impact of *ACTN4* CNI in breast cancers

**Fig. 3:**
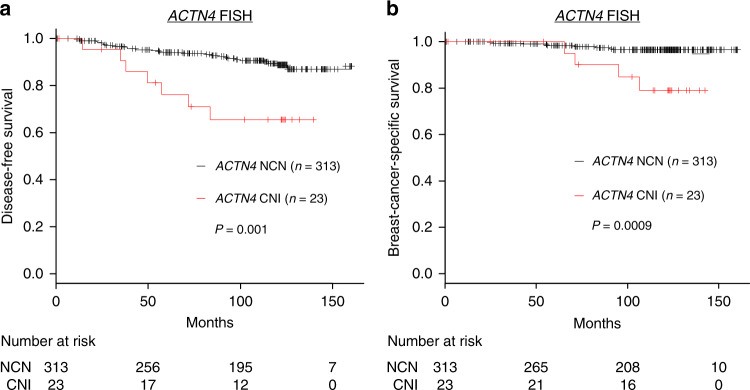
Survival curves according to the copy number of *ACTN4* (Japanese cohort). Kaplan–Meier estimate of DFS (**a**) and BCSS (**b**) of patients with HR-positive, HER2-negative, node-negative invasive breast cancer carrying (red) and not carrying (black) *ACTN4* CNI. Differences between the curves were assessed using the log-rank test. This figure has originally appeared in an abstract of the 36th annual meeting of the Japan Society for Molecular Tumor Marker Research and is reproduced with permission from the society.

*ACTN4* CNI was significantly correlated (*P* = 0.0008, log-rank test) with poor DFS in the 336 patients with HR-positive, HER2-negative, lymph node-negative invasive breast cancer (Fig. 3a). The 10-year DFS rates for patients with *ACTN4* NCN and CNI were 89.1% and 65.5%, respectively. Furthermore, BCSS of patients with CNI was significantly worse than that of patients without CNI (*P* = 0.0009, log-rank test) (Fig. 3b). The 10-year BCSS rates for patients with NCN and CNI were 96.5% and 79.1%, respectively. There were 249 stage I and 87 stage II HR-positive, HER2-negative, node-negative patients. *ACTN4* CNI was associated with significantly poor DFS and BSS in both stages (Supplementary Figs. S2 and S3).

*ACTN4* CNI was not significantly associated with poor outcome in patients with HER2-positive or triple-negative breast cancer (Supplementary Figs. S4 and S5).

### Multivariate analysis

We calculated the hazard ratios (HR) of selected parameters including patient age, menopausal status, area of invasive tumour, histology, histological grade, nuclear grade, lymphovascular invasion, Ki-67 status, and *ACTN4* copy number status for recurrence and death using univariate and multivariate Cox regression analyses. Univariate analysis revealed that histological grade (*P* = 0.006; HR, 2.52; 95% confidence interval [CI]: 1.3–4.86), nuclear grade (*P* = 0.005, HR, 2.57; 95% CI: 1.33–4.94), and *ACTN4* FISH (*P* = 0.002, HR, 3.62; 95% CI: 1.59–8.27) were significantly correlated with recurrence of breast cancer. Histological and nuclear grades are mutually correlated. Multivariate analysis revealed that the copy number status of *ACTN4* (*P* = 0.01, HR, 2.95; 95% CI: 1.27–6.83) was the sole independent risk factor for recurrence (Table 2).

**Table 2:** Cox proportional hazards model analysis of factors associated with recurrence (Japanese cohort).

Characteristics	Univariate analysis			Multivariate analysis		
	Hazard ratio	95% CI	*P* value*	Hazard ratio	95% CI	*P* value*
Age						
≤50/>50	0.97	0.50–1.89	0.81			
Menopausal status						
Pre/Post	0.85	0.44–1.63	0.62			
Area of invasive tumour						
≤2 cm/>2 cm	1.54	0.78–3.05	0.21			
Histology						
IDC/others	0.56	0.20–1.59	0.28			
Histological grade						
1–2/3	2.52	1.3–4.86	**0.006**	1.45	0.44–4.78	0.54
Nuclear grade						
1–2/3	2.57	1.33–4.94	**0.005**	1.69	0.51–5.59	0.4
Lymphovascular invasion						
Negative/Positive	0.74	0.37–1.47	0.39			
Ki67						
<14%/≥14%	1.73	0.89–3.53	0.11			
*ACTN4* FISH						
NCN/CNI	3.62	1.59–8.27	**0.002**	2.95	1.27–6.83	**0.01**

*IDC* intraductal carcinoma, *FISH* fluorescence in situ hybridisation, *NCN* normal copy number, *CNI* copy number increase, *CI* confidence interval.

*Cox regression analysis. *P* values of <0.05 are shown in bold.

Univariate analysis revealed that histological grade (*P* = 0.0004; HR, 10.16; 95% CI: 2.81–37.1), nuclear grade (*P* = 0.002; HR, 7.52; 95% CI: 2.07–27.34), and *ACTN4* copy number status (*P* = 0.0035; HR, 5.78; 95% CI: 1.78–18.77) were significantly associated with breast cancer-associated death. Histological and nuclear grades are mutually correlated. Multivariate analysis revealed that *ACTN4* copy number status (*P* = 0.014; HR, 4.27; 95% CI: 1.3–14.05) was the sole independent risk factor for death (Table 3).

**Table 3:** Cox proportional hazards model analysis of factors associated with death (Japanese cohort).

Characteristics	Univariate analysis			Multivariate analysis		
	Hazard ratio	95% CI	*P* value*	Hazard ratio	95% CI	*P* value*
Age						
≤50/>50	0.84	0.28–2.51	0.76			
Menopausal status						
Pre/Post	0.68	0.23–2.0	0.49			
Area of invasive tumour						
≤2 cm/>2 cm	2.26	0.76–6.71	0.14			
Histology						
IDC/others	0.37	0.05–2.88	0.34			
Histological grade						
1–2/3	10.16	2.81–37.1	**0.0004**	17.02	0.49–589.2	0.12
Nuclear grade						
1–2/3	7.52	2.07–27.34	**0.002**	0.5	0.01–17.48	0.7
Lymphovascular invasion						
Negative/Positive	0.91	0.3–2.77	0.86			
Ki67						
<14%/≥14%	2.74	0.84–8.9	0.093			
*ACTN4* FISH						
NCN/CNI	5.78	1.78–18.77	**0.0035**	4.27	1.3–14.05	**0.014**

*IDC* intraductal carcinoma, *FISH* fluorescence in situ hybridisation, *NCN* normal copy number, *CNI* copy number increase, *CI* confidence interval.

*Cox regression analysis. *P* values of <0.05 are shown in bold.

### Validation cohort

In order to validate the universality of the above results obtained by examining a Japanese cohort, we examined another patient cohort from a different country (the USA) using FISH. This US cohort included 261 tissue samples from patients with HR-positive, HER2-negative, node-negative invasive breast cancers. The clinicopathological characteristics of this US cohort and the correlations with *ACTN4* CNI are shown in Supplementary Table S1.

**Fig. 4:**
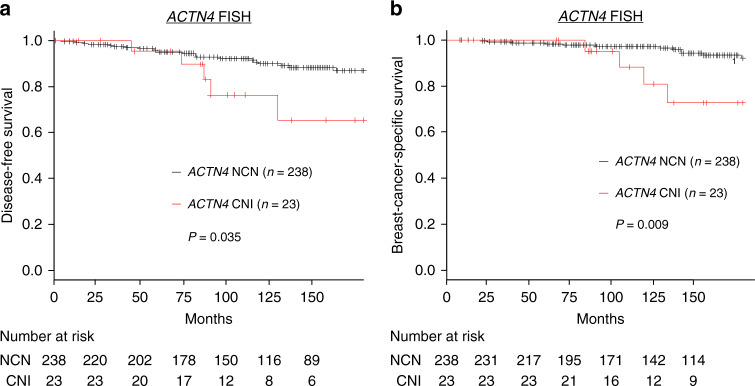
Survival curves according to the copy number of *ACTN4* (US cohort). Kaplan–Meier estimate of DFS (**a**) and BCSS (**b**) of patients with HR-positive, HER2-negative, node-negative invasive breast cancer carrying (red) and not carrying (black) *ACTN4* CNI. Differences between the curves were assessed using the log-rank test.

*ACTN4* CNI was significantly associated with histological (*P* = 0.024) and nuclear (*P* = 0.021) grades. Kaplan–Meier analysis revealed that patients with *ACTN4* CNI had a significantly poorer outcome than those with *ACTN4* NCN in terms of both DFS and BCSS (*P* = 0.035 and 0.009, respectively, log-rank test) (Fig. 4). The 10-year BCSS rates for patients with *ACTN4* NCN and CNI were 97.2% and 80.9%, respectively. Univariate Cox regression analysis showed that *ACTN4* CNI was the sole factor significantly associated with high risks of recurrence (*P* = 0.04; HR, 2.73; 95% CI 1.03–7.24) and breast cancer death (*P* = 0.016; HR, 4.01; 95% CI: 1.29–12.49) (Supplementary Tables S2 and S3).

## Discussion

HR-positive, HER2-negative, node-negative invasive breast cancers comprise a heterogeneous population, and a significant proportion of patients suffer recurrences even after complete surgical resection of their primary tumours. Therefore, it is important to establish a method of identifying high-risk patients for whom adjuvant chemotherapy would be necessary. ER and PgR status is determined by IHC, HER2 status is determined by IHC and/or FISH,^3^ and the tumour proliferative fraction is most commonly assessed by Ki-67 immunostaining, but the 15th St. Gallen International Expert Consensus on the Primary Therapy of Early Breast Cancer 2017 (St. Gallen 2017) raised an issue of caution about the reproducibility of IHC for Ki67 and its use for clinical decision-making. We previously examined the prognostic impact of 3 different Ki-67 cut-off values (10%, 14 and 20%), but no absolute cut-off value was found to reproducibly stratify patients with node-negative luminal breast cancer.^6^ St. Gallen 2017 alternatively recommended the use of multi-gene molecular assays to obtain accurate prognostic information.^3^ These multi-gene assays were reproducible across institutions and had been successfully incorporated into several clinical trials of adjuvant chemotherapy.^23–25^ However, their cost and complexity preclude their worldwide application to routine oncological practice.

In the present study, we examined the feasibility of *ACTN4* copy number status as a novel single-gene assay for stratification of node-negative luminal breast cancer. We first examined the genetic status of *ACTN4* in 336 HR (ER/I)-positive, lymph node-negative breast cancers that had been resected in Japan. The prognostic significance was then subjected to independent validation in a large cohort of patients with node-negative (Stage I) luminal breast cancer treated in the USA. The result was highly reproducible across the two countries with different racial backgrounds. The 10-year DFS rates for patients with NCN and CNI of the *ACTN4* gene were 89.1% and 65.5%, respectively, in Japan and 89.8% and 76.3%, respectively, in the USA. The rates of patients with *ACTN4* CNI seem comparable to those of high-risk patients categorised by the 21-gene recurrence score (31 or higher).^7^ The NSABP B-20 prospective-retrospective study revealed that addition of chemotherapy to tamoxifen had therapeutic benefits for patients with node-negative, ER-positive breast cancer with a 21-gene recurrence score of ≥31,^26^ indicating that patients with node-negative luminal breast cancer carrying *ACTN4* CNI might similarly benefit from postoperative adjuvant chemotherapy.

Metastasis is the major form of cancer recurrence. The actinin-4 protein is highly concentrated at the leading edges of actin-rich cell protrusions and directly regulates cell motility through remodelling of the actin cytoskeleton.^15^ It has been shown that the expression of actinin-4 in focally dedifferentiated cancer cells at the invasive front is significantly correlated with metastasis.^15^ Epithelial-mesenchymal transition (EMT) is an essential step in cancer metastasis.^27^ Actinin-4 overexpression changes cell morphology from epithelial-like to mesenchymal-like^28^ and promotes cancer cell migration and invasion through the expression of Snail and stabilised β-catenin.^29^ Therefore, molecular therapeutics targeting actinin-4 would likely inhibit cancer metastasis and cure patients with a high risk of recurrence. We recently revealed that a small-molecule Traf2 and Nck-interacting kinase (TNIK) inhibitor, NCB-0846,^30^ suppressed the EMT and metastasis of non-small cell lung cancer cells through post-translational modification of actinin-4 (unpublished observation). This compound could be applicable for prevention of recurrence in breast cancer patients with *ACTN4* CNI.

ER has been considered one of the major therapeutic targets in HR-positive breast cancer. In fact, adjuvant administration of tamoxifen to HR-positive breast cancer patients reduces the rates of recurrence and mortality.^31^ Oestrogen binds to ER in the cytoplasm and translocates into the nucleus, where it binds to DNA sequences and promotes mammary cell differentiation and proliferation. In the nucleus, actinin-4 is known to function as a co-activator of several transcriptional factors, independently from its cytoplasmic actin-binding function.^32,33^ Khurana et al. showed that overexpression of *ACTN4* activated ERα-mediated transcription activity, whereas knockdown of *ACTN4* inhibited oestrogen-mediated cancer cell proliferation.^34^ ER signalling may be activated in patients with *ACTN4* CNI and promote the aggressiveness of HR-positive tumours. Consistently, *ACTN4* CNI was not significantly associated with poor outcome in patients with HER2-positive or triple-negative breast cancer (Supplementary Figs. S4 and S5).

This study had a few limitations. The first is that it was retrospective in design. However, this study was carefully performed in accordance with the Reporting Recommendations for Tumor Marker Prognostic Studies (REMARK),^35^ and we have provided diagrams (Fig. 1 and Supplementary Fig. S1) to clearly indicate the numbers of individuals included at different stages, and incorporated into univariate and multivariable analyses. Second, the treatment of patients in the Japanese cohort was not based on a pre-fixed protocol but determined empirically by their attending physicians. Purely for this reason, we did not analyse the effects of different therapeutics in patients with and without *ACTN4* CNI. Third, we re-used TMAs that had been prepared for a previous study,^6^ and therefore 33 tissue cores (out of 369) were missing and not evaluable (Fig. 1). However, a significant number of tissue cores were found to be intact, and this random case exclusion was unlikely to have affected the overall results.

We conclude that *ACTN4* copy number analysis is a cost-effective single gene assay and can be potentially applicable for selection of high-risk patients with HR-positive, HER2-negative, lymph node-negative invasive breast cancer. Its prognostic impact is significant and reproducible, warranting further independent validation and clinical trials by other investigators.

## Supplementary information

SUPPLEMENTAL MATERIAL

## Data Availability

The datasets generated and/or analysed during the present study are not publicly available, but anonymised versions may be available from the corresponding author on reasonable request.
