# Supplementary material for: Prognostic impact of ACTN4 gene copy number alteration in hormone receptor-positive, HER2-negative, node-negative invasive breast carcinoma
Source: Br J Cancer. 2020 Apr 8;122(12):1811–7. doi: 10.1038/s41416-020-0821-y (PMC7283275; doi:10.1038/s41416-020-0821-y)
Supplement: Supplementary file 1 — SUPPLEMENTAL MATERIAL [file 41416_2020_821_MOESM1_ESM.pdf]

Supplementary Table S1. Correlation of *ACTN4* gene status with clinicopathological characteristics (US cohort).

Characteristics	Total	*ACTN4* FISH		*P* value*
		NCN	CNI	
**Total**	261	238	23	
**Age**				
≤ 50	61	58	3	0.304
> 50	200	180	20	
**Histology**				
Invasive ductal carcinoma	235	213	22	0.487
Others	26	25	1	
**Histological grade**				
1 or 2	233	216	17	**0.024**
3	28	22	6	
**Nuclear grade**				
1 or 2	226	210	16	**0.021**
3	35	28	7	
**Adjuvant chemotherapy**				
Yes	24	23	1	0.705
No	237	215	22	
**Hormone therapy**				
Yes	87	75	12	0.062
No	174	163	11	

Abbreviations: FISH, fluorescence *in situ* hybridization; NCN, normal copy number; CNI, copy number increase.

*Fisher's exact test. *P* values of < 0.05 are shown in bold.

Supplementary Table S2. Cox proportional hazards model analysis of factors associated with recurrence (US cohort)**.**

Characteristics	Univariate analysis			Multivariate analysis		
	Hazard Ratio	95% CI	*P* value*	Hazard Ratio	95% CI	*P* value*
**Age**						
≤ 50/> 50	0.77	0.34-1.76	0.53			
**Histology**						
IDC/others	1.12	0.34-3.71	0.86			
**Histological grade**						
1-2/3	1.34	0.46-3.89	0.59			
**Nuclear grade**						
1-2/3	1.07	0.37-3.09	0.9			
***ACTN4* FISH**						
NCN/CNI	2.73	1.03-7.24	**0.04**			

Abbreviations: IDC, intraductal carcinoma; FISH, fluorescence *in situ* hybridization; NCN, normal copy number; CNI, copy number increase; CI, confidence interval.
*Cox regression analysis. *P* values of < 0.05 are shown in bold.

Supplementary Table S3. Cox proportional hazards model analysis of factors associated with death (US cohort)**.**

Characteristics	Univariate analysis			Multivariate analysis		
	Hazard Ratio	95% CI	*P* value*	Hazard Ratio	95% CI	*P* value*
**Age**						
≤ 50/> 50	0.7	0.24-2.00	0.5			
**Histology**						
IDC/others	1.36	0.31-5.99	0.68			
**Histological grade**						
1-2/3	1.87	0.53-6.58	0.33			
**Nuclear grade**						
1-2/3	1.45	0.41-5.11	0.56			
***ACTN4* FISH**						
NCN/CNI	4.01	1.29-12.49	**0.016**			

Abbreviations: IDC, intraductal carcinoma; FISH, fluorescence *in situ* hybridization; NCN, normal copy number; CNI, copy number increase; CI, confidence interval.

*Cox regression analysis. *P* values of < 0.05 are shown in bold.

Supplementary Figure S1

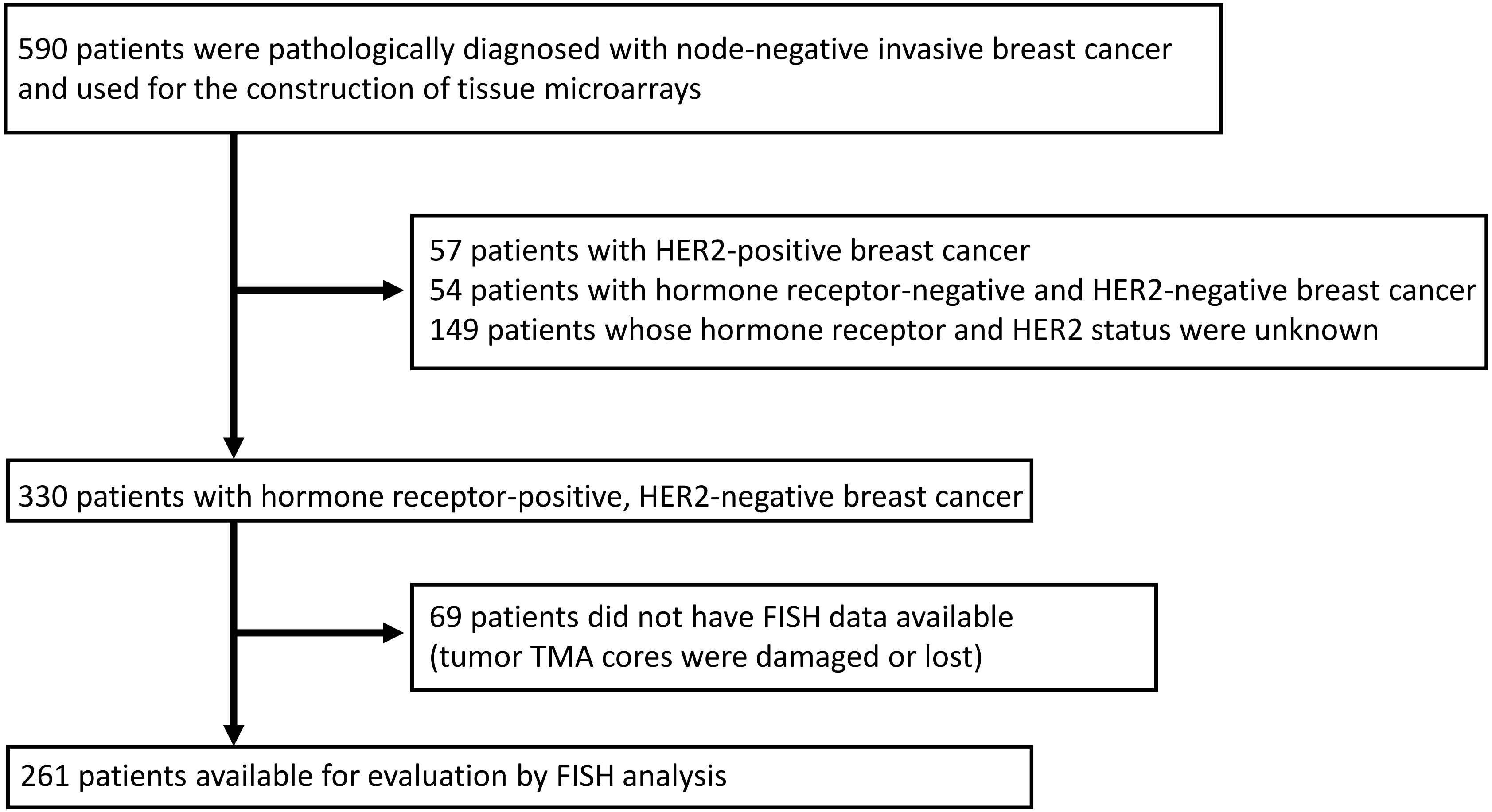

Supplementary Figure S2

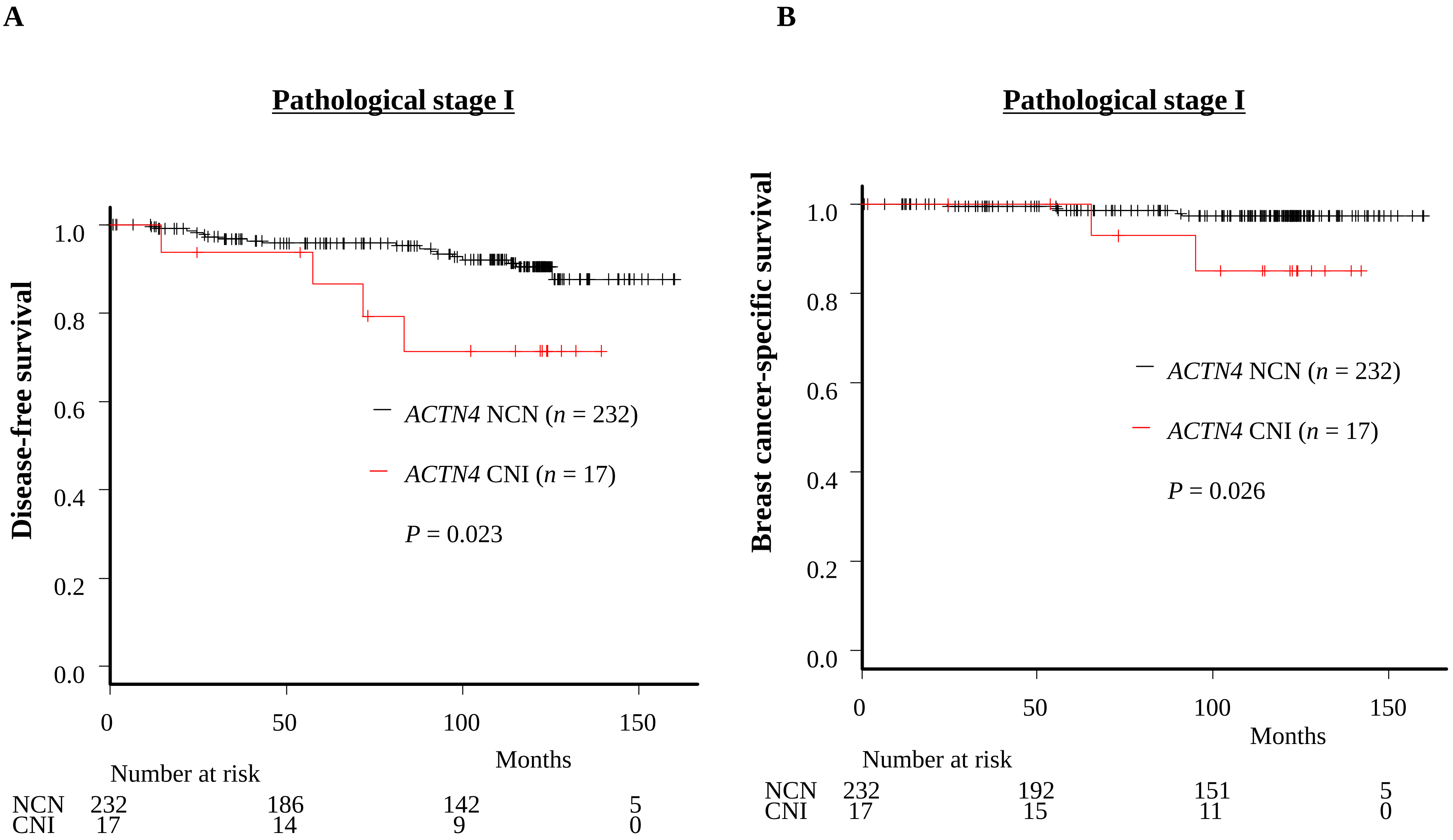

Supplementary Figure S3

A

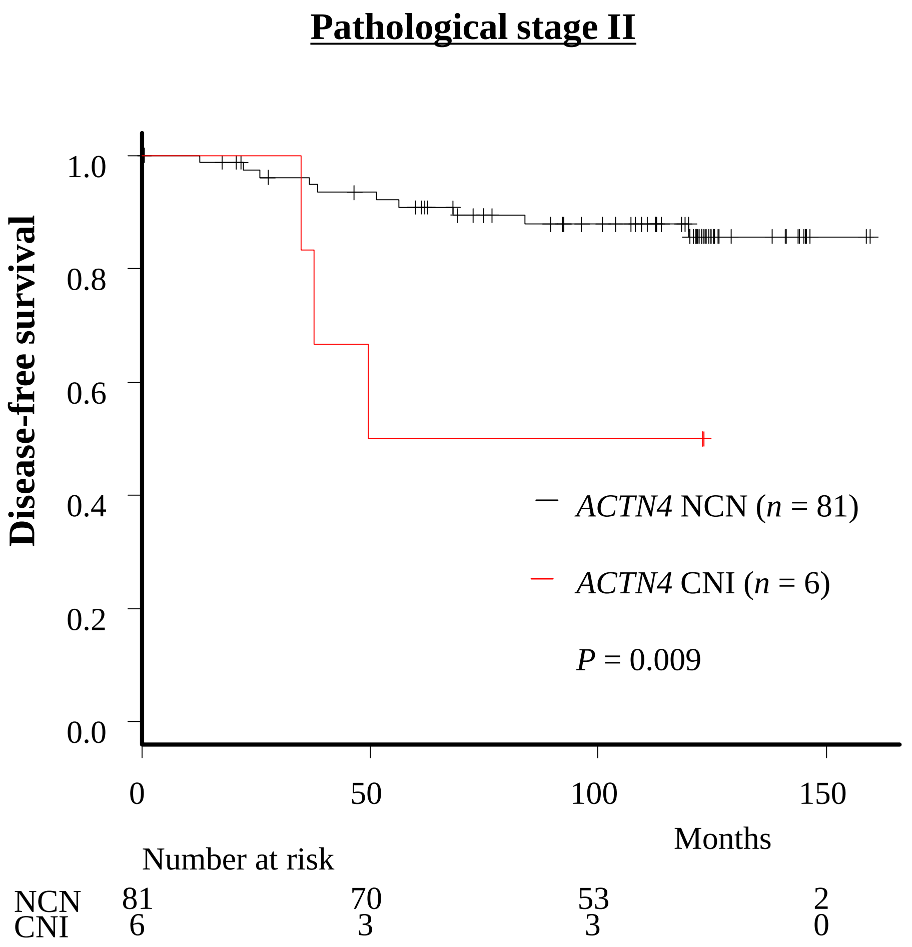

B

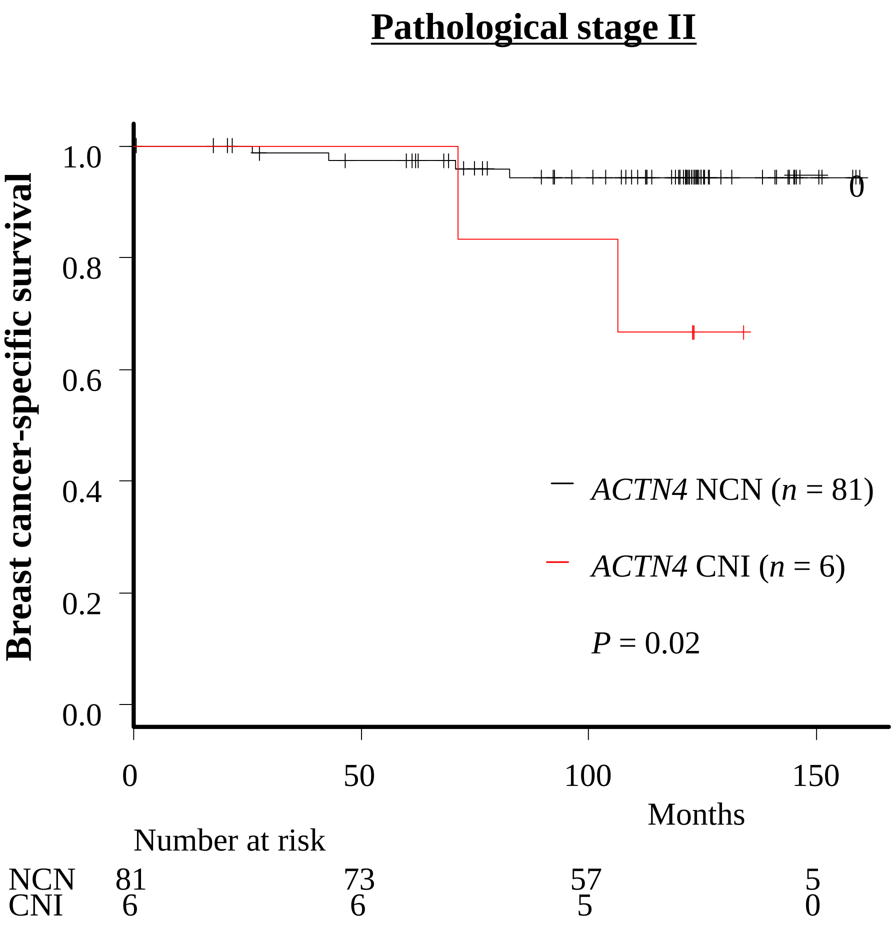

Supplementary Figure S4

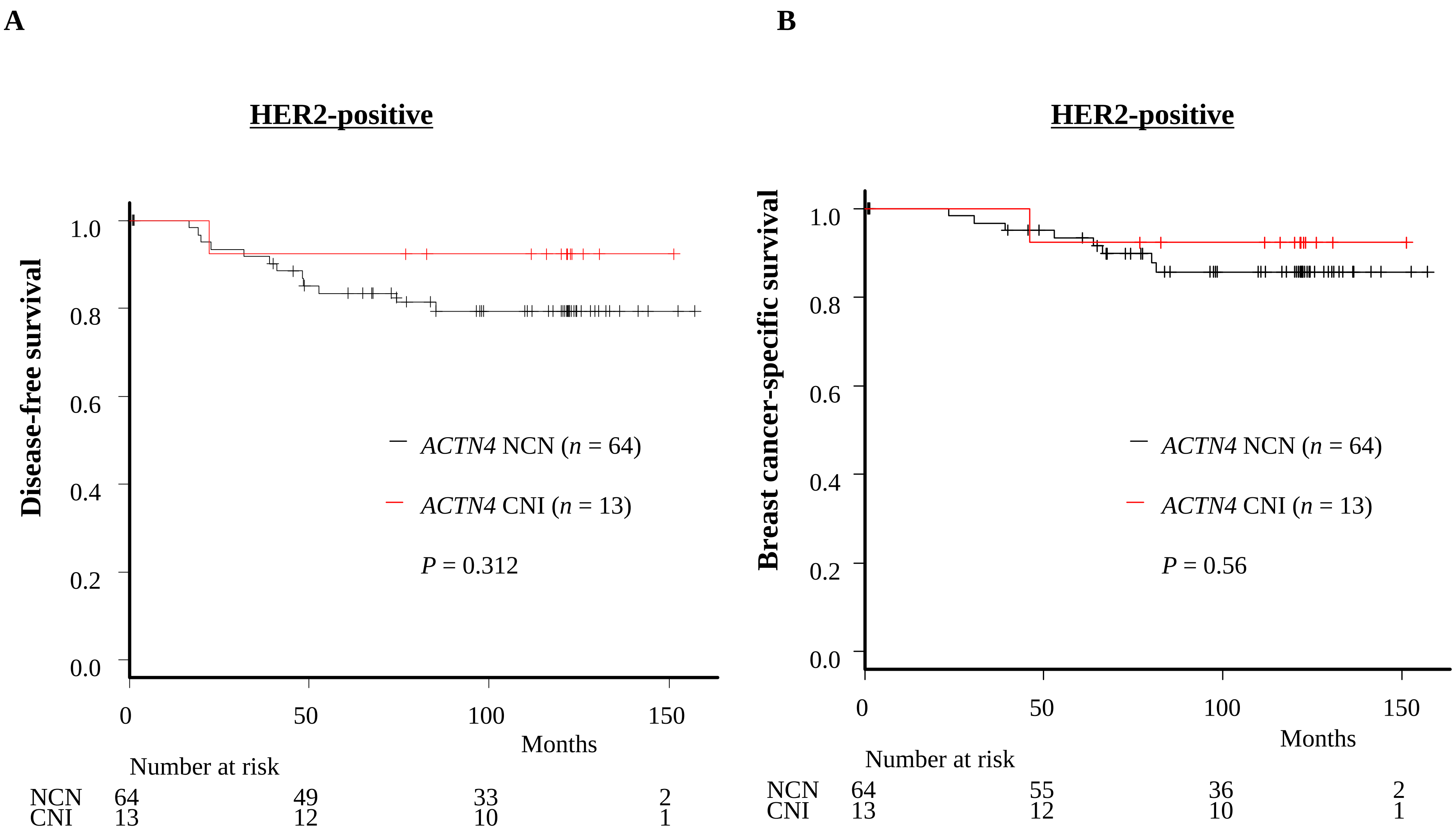

Supplementary Figure S5

A

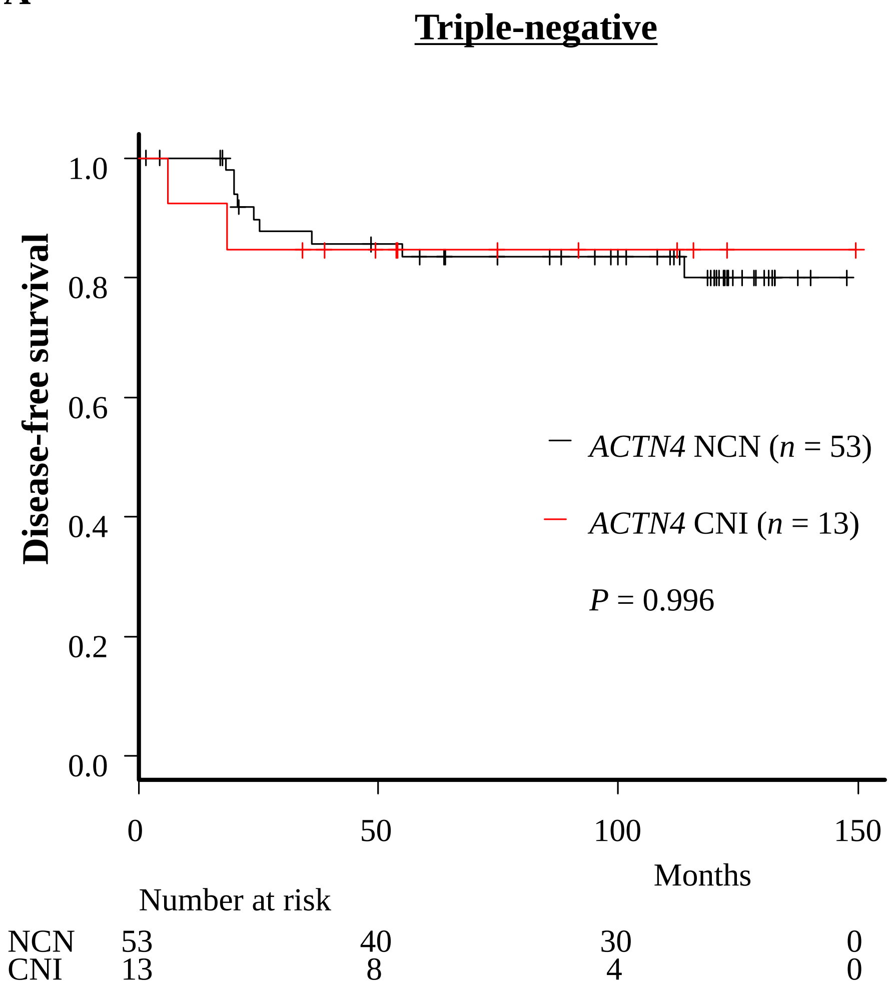

B

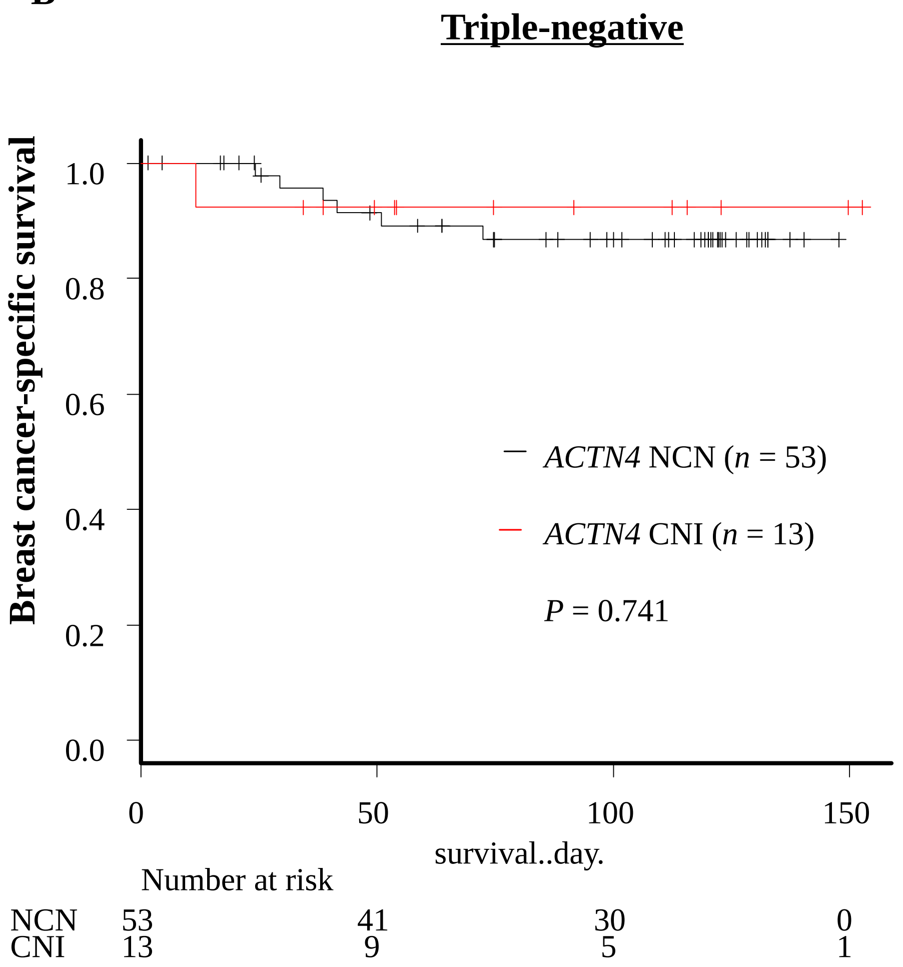

Legends for Supplementary Figures

Supplementary Figure S1. Selection of eligible individuals (US cohort)**.**

Supplementary Figure S2. Survival curves for stage I patients according to the copy number of *ACTN4* (Japanese cohort)**.**

Kaplan-Meier estimation of DFS (**A**) and BCSS (**B**) of patients with HR-positive, HER2-negative, node-negative pathological stage I invasive breast cancer carrying (red) and not carrying (black) *ACTN4* CNI. Differences between the curves were assessed using the log-rank test.

Supplementary Figure S3. Survival curves for stage II patients according to the copy number of *ACTN4* (Japanese cohort)**.**

Kaplan-Meier estimation of DFS (**A**) and BCSS (**B**) of patients with HR-positive, HER2-negative, node-negative pathological stage II invasive breast cancer carrying (red) and not carrying (black) *ACTN4* CNI. Differences between the curves were assessed using the log-rank test.

Supplementary Figure S4. Survival curves of patients with HER2-positive tumors according to the copy number of *ACTN4* (Japanese cohort)**.**

Kaplan-Meier estimation of DFS (**A**) and BCSS (**B**) of patients with HER2-positive, node-negative invasive breast cancer carrying (red) and not carrying (black) *ACTN4* CNI. Differences between the curves were assessed using the log-rank test.

Supplementary Figure S5. Survival curves of patients with triple-negative tumors

according to the copy number of *ACTN4* (Japanese cohort)**.**

Kaplan-Meier estimation of DFS (**A**) and BCSS (**B**) of patients with triple-negative, node-negative invasive breast cancer carrying (red) and not carrying (black) *ACTN4* CNI. Differences between the curves were assessed using the log-rank test.
